# The Role of HuR in the Post-Transcriptional Regulation of Interleukin-3 in T Cells

**DOI:** 10.1371/journal.pone.0092457

**Published:** 2014-03-21

**Authors:** José A. González-Feliciano, Marimar Hernández-Pérez, Luis A. Estrella, Daisy D. Colón-López, Armando López, Marina Martínez, Kirla R. Maurás-Rivera, Clarivel Lasalde, Daviana Martínez, Félix Araujo-Pérez, Carlos I. González

**Affiliations:** 1 University of Puerto Rico-Río Piedras, Department of Biology, College of Natural Sciences, San Juan, Puerto Rico; 2 Department of Biochemistry, University of Puerto Rico-Medical Sciences, San Juan, Puerto Rico; 3 Molecular Sciences Research Building, San Juan, Puerto Rico; Colorado State University, United States of America

## Abstract

Human Interleukin-3 (IL-3) is a lymphokine member of a class of transiently expressed mRNAs harboring Adenosine/Uridine-Rich Elements (ARE) in their 3' untranslated regions (3'-UTRs). The regulatory effects of AREs are often mediated by specific ARE-binding proteins (ARE-BPs). In this report, we show that the human IL-3 3'-UTR plays a post-transcriptional regulation role in two human transformed cell lines. More specifically, we demonstrate that the hIL-3 3'-UTR represses the translation of a luciferase reporter both in HeLa and Jurkat T-cells. These results also revealed that the hIL-3 3'-UTR-mediated translational repression is exerted by an 83 nt region comprised mainly by AREs and some non-ARE sequences. Moreover, electrophoretic mobility shift assays (EMSAs) and UV-crosslinking analysis show that this hIL-3 ARE-rich region recruits five specific protein complexes, including the ARE-BPs HuR and TIA-1. HuR binding to this ARE-rich region appears to be spatially modulated during T-cell activation. Together, these results suggest that HuR recognizes the ARE-rich region and plays a role in the IL-3 3'-UTR-mediated post-transcriptional control in T-cells.

## Introduction

Interleukin-3 (IL-3) is a pleiotropic cytokine that promotes the proliferation, survival and differentiation of multiple hematopoietic cell types [1]–[3]. Aberrant expression of IL-3 is associated with angiogenesis, chronic inflammation and cancer [4]–[7]. IL-3 is over-expressed in the myelomonocytic leukemia cell line WEHI-3B and in multiple myeloma patients [8], [9]. IL-3 over-expression in chronic myelogenous leukemia (CML) patients has also been associated with imatinib resistance [10]. While the role of IL-3 in cancer is unclear, accumulating evidence suggests that IL-3 is involved in inflammatory and tumor angiogenesis [7], [11].

IL-3 expression is restricted to T-lymphocytes and is regulated at the transcriptional level [12], [13]. Besides its transcriptional regulation, IL-3 is also controlled at the post-transcriptional level [13]. Interestingly, IL-3 mRNA is accumulated following T-cell activation with antigens, mitogens and phorbol esters [13], [14]. Furthermore, Adenosine/Uridine-Rich Elements (AREs) present in the 3'-UTR of the murine IL-3 (mIL-3) mRNA play a role in the post-transcriptional regulation of IL-3 during T-cell activation [15].

AREs are sequences of 50 to 150 nucleotides located in the 3'-UTRs of growth factors, cytokines and proto-oncogenes mRNAs [16], [17]. Approximately 7% of human genes encode ARE-containing mRNAs [18]. ARE-mediated post-transcriptional control is exerted by ARE-binding proteins (ARE-BPs) that can positively or negatively influence mRNA stability and/or translation [19]. For example, the ARE-BPs Tristetraprolin and butyrate response factor 1 promote mRNA turnover; whereas, HuR controls both mRNA turnover and translation [20]–[22]. Moreover, T-cell intracellular antigen 1 (TIA-1) and CUG triplet repeat binding protein have been associated with translational silencing [23], [24]. Despite the identification of various ARE-BPs that affect the rate of translation and/or mRNA turnover, it is unclear which ARE-BPs bind to specific AREs and how these interactions influence post-transcriptional control of ARE-containing mRNAs.

Previous studies have been primarily targeted towards understanding the post-transcriptional regulation mediated by the murine IL-3 ARE [15], [25], [26]. More recent bioinformatics analysis, however, have suggested that ARE cluster variations among species may have important biological consequences [18]. In addition, IL-3 is a species-specific cytokine in which the hIL-3 fails to support the proliferation of murine cells [27], [28]. Therefore, it is critical to elucidate the functional relevance of the human IL-3 (hIL-3) ARE in order to better understand its role in post-transcriptional control. Moreover, the RNA binding proteins that recognize the hIL-3 mRNA and their biological significance in the ARE-mediated control of IL-3 expression in T-cells remain elusive.

In this study, we show that the ARE-rich region within the hIL-3 3'-UTR represses the translation of a luciferase reporter in HeLa and Jurkat T-cells. We also demonstrate that the region within the hIL-3 3'-UTR that harbors several AREs recruits five specific protein complexes from 34 to 88 kDa, including the ARE-BPs HuR and TIA-1. HuR binding to the hIL-3 ARE-rich sequence appears to be spatially modulated during T-cell activation. Moreover, siRNA knockdown of HuR in T cells affects the expression of the heterologous reporter harboring the hIL-3 3'-UTR. Collectively, our results suggest that HuR specifically recognizes an ARE-rich region and plays an important role in the post-transcriptional regulation mediated by the hIL-3 3'-UTR in T-cells. In addition, the results presented in this report provide a foundation for future studies to determine the precise role of the ARE-mediated post-transcriptional pathway in the regulation of hIL-3 in resting and activated T-cells.

## Materials and Methods

### Cell culture

HeLa and Jurkat T-lymphocyte cell lines were purchased from the American Type Culture Collection (Manassas, VA, USA). HeLa cells were cultured in Dulbecco’s modified Eagle’s medium (Sigma-Aldrich) and Jurkat cells in RPMI 1640 (HyClone). Cell culture medium was supplemented with 10% fetal bovine serum, 100 U/ml penicillin, 100 μg/mL streptomycin and 250 ng/ml amphotericin B (Cellgro) at 37°C with 5% CO_2_. Jurkat cells were cultured at 1×10^6^/ml in RPMI 1640/10% FBS at 37°C and activated during 0, 6, 12 and 24 hrs with phorbol-12-myristate-13-acetate (PMA) and/or Ionomycin (IO) (Sigma-Aldrich) at 20 ng/ml and 800 ng/ml, respectively.

### Plasmids

The hIL-3 3'-UTR encompassing nucleotides 513 to 924 was amplified from Jurkat cells total RNA (Stratagene) treated with IO and PMA using reverse transcriptase polymerase chain reaction (RT-PCR). Subsequently, the hIL-3 3'-UTR cloned form Jurkat cells was compared with the human IL-3 mRNA sequence storage in NCBI (NM_000588). Sequence alignment results demonstrated that the hIL-3 3'UTR cloned from Jurkat cells is identical to the human IL-3 3'-UTR reported by Otsuka et al., 1988 [29]. In order to construct the mammalian expression vectors, the hIL-3 PCR products were digested with *XbaI*/*EcoRI* and the hIL-3 3'-UTR was cloned into pSP-luc+ vector (Promega). This recombinant plasmid was digested with *KpnI*/*EcoRI* to assemble the firefly luciferase hIL-3 3'-UTR chimeras. The luciferase chimeras were cloned into the mammalian expression vector PCDNA 3.1 + (Invitrogen) at *KpnI*/*EcoRI* sites. A firefly luciferase chimera control lacking the hIL-3 3'-UTR was modified by digesting pSP-Luc+ with *KpnI*/*XbaI*, and cloning into pCDNA 3.1+. For the hIL-3 3'-UTR ΔARE construct, the method established by Makarova *et al*. [30] was used. The IL-3 ARE motif (IL-3 3'-UTR *ΔARE)* was replaced with an 82 nucleotides stuffer sequence derived from the yeast *NPL3* gene (nts 738-819), a region lacking AUUUA sequences, using a PCR insertion protocol [31].

For *in vitro* transcription studies, the hIL-3 3'-UTR was divided into three segments: (1) a region that comprises 83nts with the putative AREs (nts 719–801); (2) a sequence of 90nts upstream of the hIL-3 ARE (nts 629–718); (3) a region encompassing 85nts downstream of the hIL3-ARE (nts 802–886). The IL-3 3'-UTR upstream (UP), ARE and downstream (Down) regions were cloned using complementary DNA oligos flanked by *XbaI*/*EcoRI* restriction sites and cloned into the pSP-Luc+ vector. To eliminate the luciferase reporter from these constructs, plasmids were digested with *XbaI*/*KpnI*. The pGEM-7Z+ (Promega) vector linearized with *EcoRI* was used to generate the 80nts control RNA (c-RNA).

### Cell transfections and Luciferase reporter gene assays

Jurkat and HeLa cells were transfected following manufacturer’s instructions with Fugene HD (Roche) and Lipofectamine LTX (Invitrogen), respectively. For both cell lines, 4.0 μg of firefly and 0.4 μg of *Renilla* luciferase plasmids were used. After transfection, cells were incubated for 48 hrs at 37°C and luciferase activities were measured using the dual luciferase assay kit (Promega). Plasmids and siRNAs were co-transfected using electroporation. Prior to electroporation, Jurkat cells were washed twice with PBS and resuspended in RPMI 1640 at a density of 3×10^7^/ml. Cell suspensions (300 μl) were added to 4-mm gap electroporation cuvettes (BTX); DNA (20 μg firefly and 5 μg *Renilla* luciferase) and 10 μl of HuR 20 μM siRNA (Dharmacon Res., Inc.) were diluted into 100 μl of Opti-MEM I. A non-targeting siRNA was used as control. DNA/siRNA mixtures were added to the cuvettes and subjected to a single pulse at 310 V for 10ms in an ECM 830 square-wave electroporator (BTX). Cells were plated on 6-well plates and cultured in complete medium without antibiotics for 48 hrs. After cell harvesting, luciferase activities were measured and protein samples obtained for HuR immunoblotting.

### In vitro transcription

Plasmids containing control, UP, ARE and Down regions were linearized with *EcoRI*. Plasmids were *in-vitro* transcribed in the presence of radionucleotides (^32P^UTP) using SP6 RNA polymerase (Promega). RNA loading dye (95% formamide, 0.025% xylene cyanol, 0.025% bromophenol blue, 18 mM EDTA and 0.025 % SDS) was added; samples were boiled for 5 min and loaded onto a 5% polyacrylamide 7 M urea gel with 1X TBE running buffer. The gel was exposed to X-ray film (Kodak) and bands corresponding to the RNA probes were excised. Each gel slice was incubated at 25°C overnight with 500 μl of elution buffer (300 mM sodium acetate, 50 mM Tris-Cl [pH 8.0], 5 mM EDTA and 1% SDS) containing 20 μg of proteinase K. After elution, RNAs were extracted twice with phenol:choloroform (5:1) and precipitated with 100% ethanol. RNA pellets were washed with 70% ethanol and air dried. All samples were resuspended with H_2_O and quantified by scintillation counting.

### Cell extracts

Cytosolic and nuclear extracts were prepared using the method established by Mukherjee *et al* [32] and Okada *et al* [33], respectively. Separation of the nuclear fraction from the cytosol was verified by immunoblotting with β-actin (cytoplasmic) and hnRNP C1/C2 (nuclear).

### Electrophoretic mobility shift assays (EMSAs)

EMSAs were carried out following the method of Kandasamy *et al*. [34] with minor modifications. A total of 1×10^5^ cpm of control RNA, UP, ARE and Down ^32^P-UTP labeled hIL-3 3'UTR regions were incubated with 5–10 μg of cytoplasmic HeLa and Jurkat protein extracts in 20 μl reaction mixtures with 20 units of RNasin and 1X EMSA buffer (15 mM HEPES [pH 7.9], 15 mM KCl, 5 mM MgCl_2_, 2 mM dithiothreitol, 0.1 mM spermidine, 5 mg/ml heparin, 1 mg/ml yeast tRNA). EMSA reactions were incubated for 10 min at 25°C followed by incubation on ice for 20 min. EMSA super shifts were performed by adding 1 μg of TIA-1 (C-20, goat polyclonal IgG), TIAR (C-18, goat polyclonal IgG) and HuR (3A2, mouse polyclonal IgG) antibodies from Santa Cruz Biotechnology and AUF-1 (07-260, rabbit polyclonal) antibody from Upstate Biotechnology. As a negative control, an anti-goat IgG (sc-2350) secondary antibody from Santa Cruz Biotechnology was used. After antibody addition, reactions were incubated for 15 min on ice; 15 μl of EMSA loading dye (10% sucrose in 50 mM Tris-HCl ph 7.5) were added and reaction mixtures were resolved by electrophoresis under non-denaturing conditions in 5% polyacrylamide gels with 45 mM TBE running buffer. Gels were then vacuum-dried and analyzed by phosphorimaging (BioRad Molecular Imager FX).

### UV cross-linking assays

A total of 1×10^5^ cpm of control RNA, UP, ARE and Down ^32^P-UTP labeled hIL-3 3'-UTR regions were incubated for 30 min with 5 μg of cytoplasmic protein extracts from HeLa and Jurkat cells as described for EMSAs. Upon incubation, samples were treated with 30 mJ of UV light for 15 min. Reactions were treated with RNase A to a final concentration of 0.1 μg/μl for 30 minutes at 30°C. To a similar set of reactions, 15 μg of proteinase K were added and incubated for 30 min at 37°C. Samples were run in a 10% SDS-PAGE gel and analyzed by phosphorimaging.

### Quantitative RT-PCR

Total RNA from transiently transfected HeLa and Jurkat cells was isolated using TRIzol (Invitrogen) following manufacturer’s recommendations. DNA contamination was prevented with Turbo DNA-Free kit (Applied Biosystems). For mRNA quantification, 1 μg of total RNA from HeLa and Jurkat cells was converted to cDNA using the iScript cDNA synthesis kit (BioRad). Quantitative RT-PCR was performed with the iQ SYBR Green Super Mix and 0.1 μg of cDNA. To amplify IL-3, GAPDH, firefly and *Renilla* luciferases the following primers were used: IL-3 forward 5'-CTT TGC CTT TGC TGG ACT TCA ACA A-3', IL-3 reverse 5'-GCA GAC ATG GCA GGA GAT TTT TAA G-3', GAPDH forward 5'-GGT GAA GGT CGG AGT CAA CGG A-3', GAPDH reverse GAG GGA TCT CGC TCC TGG AAG A-3', firefly luc forward 5'-ATG AAA CGA TAT GGG CTG AA-3', firefly luc reverse 5'-TTT TTG GAA ACG AAC ACC AC-3', *Renilla* luc forward 5'-TAT GGG CAA ATC AGG CAA AT-3' and *Renilla* luc reverse 5'-CCA TTC ATC CCA TGA TTC AA-3'. Samples were analyzed in the Mastercycler Realplex^2^ from Eppendorf and mRNA levels estimated using the 2^−*ΔΔ*Ct^ statistical analysis.

### Western Blots

Jurkat cytoplasmic, nuclear and total protein extracts were separated in 10% SDS-PAGE and transferred to nitrocellulose membranes that were incubated with HuR and hnRNP C1/C2 antibodies. β-actin (Abcam) immunoblotting was used as a loading control and HuR levels were normalized against β-actin levels.

### Statistical and bioinformatics analysis

Values represent mean ± standard error of the mean from three independent transfections. Differences in mean between experimental groups were analyzed by Student’s unpaired *t*-test and p<0.05 was considered statistically significant.

## Results

### The ARE-rich region within the hIL-3 3'-UTR regulates the expression of a heterologous luciferase reporter in HeLa and Jurkat T-cells

**Figure 1:**
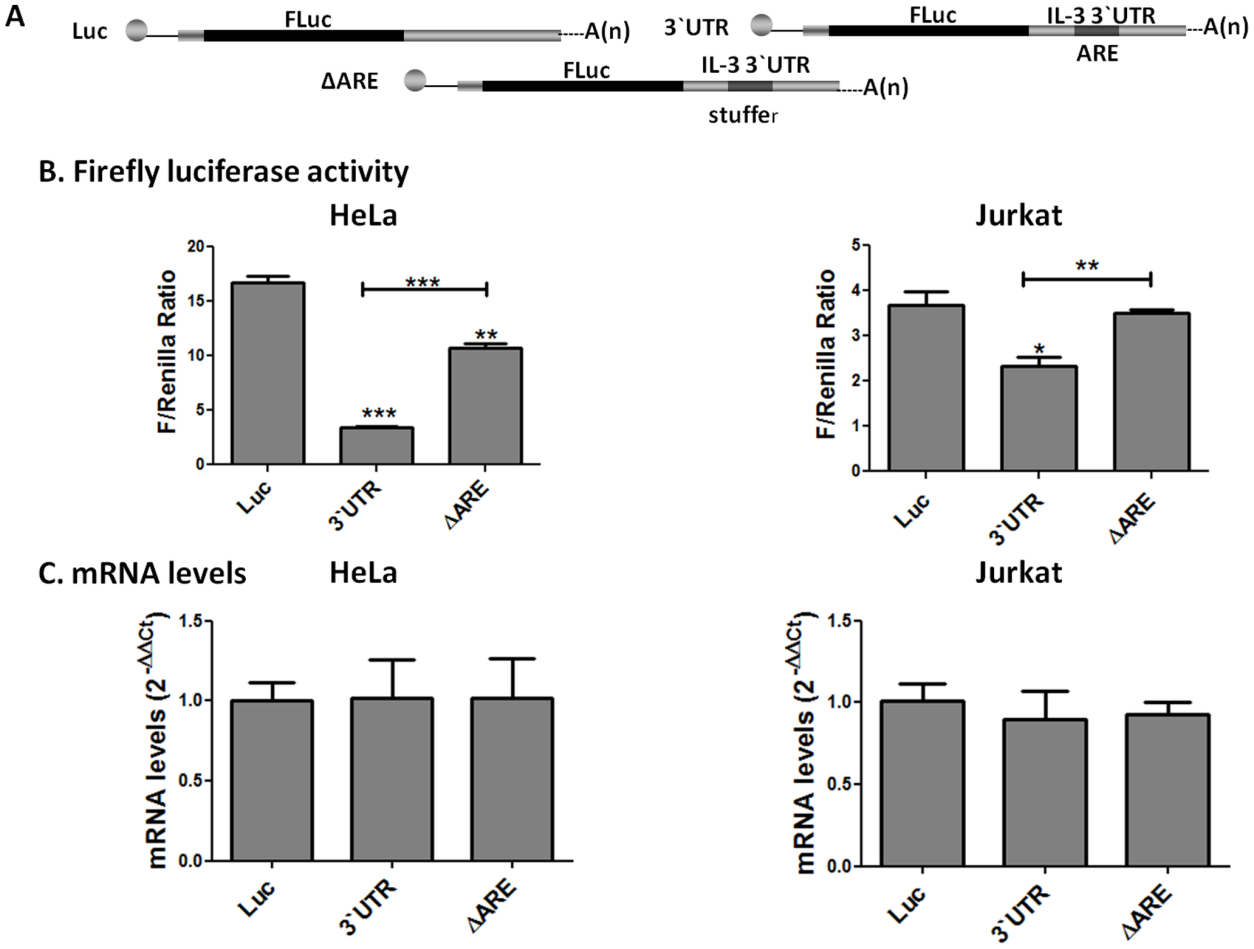
Human IL-3 ARE-rich region acts as a translational repressor element. (A) Schematic representation of the firefly luciferase reporter constructs. The human IL-3 3'-UTR was cloned into a heterologous firefly luciferase reporter. The IL-3 ARE-rich region was deleted from the 3'-UTR and replaced with a stuffer sequence (ΔARE). A firefly chimera without the hIL-3 3'-UTR (Luc) was used as a control. HeLa and Jurkat cells were transfected with the chimeric reporter constructs Luc, 3'-UTR, and ΔARE. Forty-eight hours post-transfection, (B) luciferase activity and (C) mRNA levels were measured. RNA levels were determined by Real Time RT-PCR. Triplicates were normalized using *Renilla* luciferase as an internal transfection control. Values represent mean ± standard error of the mean (SEM) from three independent transfections. Two-tailed *t*-tests were used for statistical analysis. The asterisk indicates a statistical significant (P<0.05) result when compared to the Luc control.

The 3'-UTRs of various cytokines (e.g. IL-2 and TNF-α) and proto-oncogenes (e.g. *c-myc* and *c-fos*) harbor specific *cis-*acting elements, such as AREs, previously implicated in the control of gene expression by regulating mRNA stability and/or translation [16], [17]. Sequence analysis revealed the presence of an ARE-rich region within the 3'-UTR of the human IL-3 transcript (data not shown). This 83 nt sequence designated as the ARE-rich region is comprised mainly by AREs (81%) and some non-AREs sequences (19%). To elucidate the role of this hIL-3 ARE-rich region, heterologous reporter constructs were designed, in which the entire sequence encoding the hIL-3 3'-UTR or the hIL-3 3'-UTR deleted for the ARE-rich region and replaced with a stuffer DNA sequence of similar size (ΔARE) were inserted downstream of the firefly luciferase coding region (**Fig. 1A**). Dual luciferase assays were carried out at 48 hours after transient transfection of the chimeric constructs into HeLa cells. Previous studies have employed HeLa cells to characterize the functional relevance of the ARE-mediated post-transcriptional regulatory pathway of various cytokines [35], [36]. As shown in the left panel of **Fig. 1B** **,** luciferase activity was reduced approximately 6-fold in cells transfected with the vector containing the entire hIL-3 3'-UTR compared with cells transfected with luciferase alone (Luc). In contrast, the decrease in luciferase activity was markedly attenuated in cells transfected with the reporter harboring the hIL-3 3'-UTR deleted for the ARE-rich region. Upon examination of mRNA steady-state levels of the chimeric reporters by quantitative RT-PCR assays, we did not observe significant differences in mRNA abundance between the luciferase control, the hIL-3 3'-UTR and ΔARE heterologous reporters (**Fig. 1C** **).** These results suggest that the hIL-3 3'-UTR acts as a negative regulator of luciferase expression in HeLa cells and this inhibition is influenced by the ARE-rich region.

To further evaluate the hIL-3 3'-UTR-mediated repression of luciferase activity in cells that endogenously express IL-3, the luciferase reporter constructs were also transfected into Jurkat T-cells. Dual luciferase assays demonstrated that the hIL-3 3'-UTR represses the luciferase activity approximately 2-fold compared with T-cells transfected with luciferase alone. Moreover, this inhibitory effect is abolished by deletion of the ARE-rich region as previously observed in HeLa cells (**Fig. 1B**, right panel). Quantification of mRNA steady-state levels showed no significant differences between the luciferase control, the hIL-3 3'-UTR and ΔARE heterologous reporters (**Fig. 1C,** right panel). These results suggest that the ARE-rich region within the hIL-3 3'-UTR regulates luciferase expression without affecting mRNA steady-state levels of the chimeric reporters in both HeLa and Jurkat T-cells. Furthermore, these data imply that the hIL-3 ARE-rich region may act as a translational repressor element in these human cancer cell lines under the conditions assayed in our analysis.

### The hIL-3 ARE-rich region is recognized by specific RNA binding protein complexes

**Figure 2:**
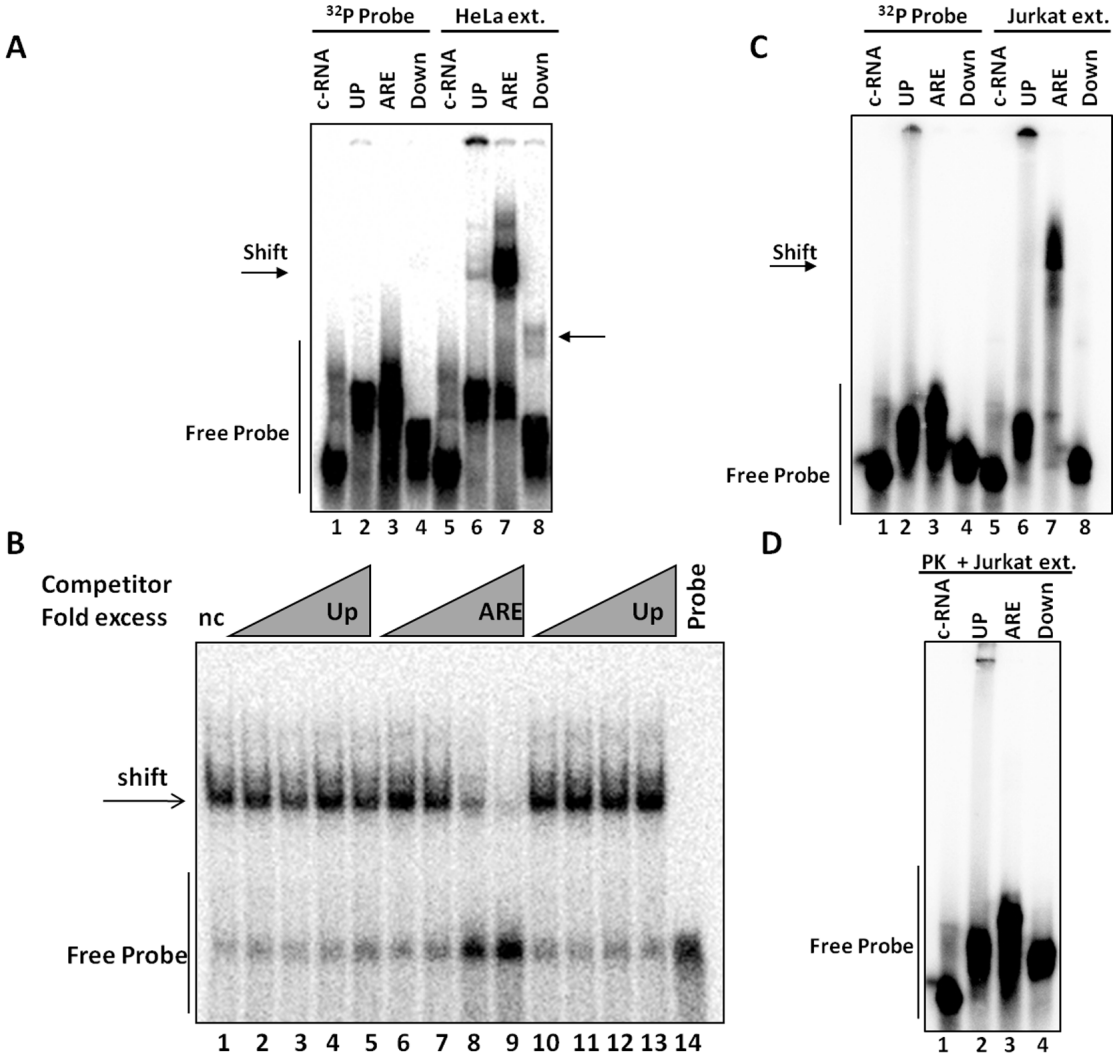
The hIL-3 ARE-rich region is recognized by specific RNA-binding protein complexes in both HeLa and Jurkat T-cells. (A) Radiolabeled RNA probes corresponding to the hIL-3 3'-UTR UP, ARE and Down regions were incubated with (lanes 5–8) or without (lanes 1-4) cytoplasmic extracts from HeLa cells. Arrows indicate the obtained gel shifts. (B) To assess the specificity of the RBP complexes that recognize the IL-3 ARE-rich region in HeLa cells, an EMSA competition assay was performed using cold RNA competitors at increasing fold-excess (10^1^–10^4^): Up (lanes 2–5), ARE (lanes 6–9) or Down (lanes 10–13). Non- competitor RNA (nc) was added in lane 1. The pGem7z multiple cloning site (80nt) was used as a negative control RNA (c-RNA). (C) EMSAs were also performed using cytoplasmic extracts from Jurkat cells. (D) EMSA reactions were treated with 15 μg of Proteinase K (PK).

Previous studies have demonstrated that the mRNA stability and/or translational effects mediated by AREs rely on their interactions with ARE-BPs [16], [17]. Wang *et al.* showed that several RNA binding proteins recognize the murine IL-3 ARE [25]. At present, little is known about the RNA binding proteins (RBPs) that recognize and modulate IL-3 expression in humans. To determine whether specific ARE-BP complexes recognize the ARE-rich RNA sequence within the hIL-3 3'-UTR, electrophoretic mobility shift assays (EMSAs) were conducted with Jurkat cell extracts. For these EMSAs, the entire hIL-3 3'UTR was divided into three segments: 1) a region 90 nucleotides upstream (Up) of the ARE; 2) 83 nucleotides encompassing the hIL-3 ARE-rich region; and 3) 85 nucleotides downstream (Down) of the ARE. As a negative control, a non-ARE containing RNA (c-RNA) consisting of 80 nucleotides from the pGem7z multiple cloning site was used. These radiolabeled *in vitro* transcribed RNAs were incubated with HeLa cytoplasmic protein extracts and analyzed by EMSAs. As shown in **Fig. 2A** **,** a predominant shift is observed in the presence of the hIL-3 ARE RNA (lane 7). In contrast, the binding activities observed for RNAs corresponding to the Up and Down IL-3 regions were weaker in nature (lanes 6 and 8). Importantly, no RBP complexes were detected in the presence of the control-RNA (lane 5). Furthermore, an EMSA competition assay demonstrated the specificity of the RBPs that recognize the IL-3 ARE sequence since it is eliminated by the addition of excess unlabeled hIL-3 ARE (**Fig. 2B**, lanes 6–9), but it is not altered by hIL-3 Up or Down competitor sequences (lanes 2–5 and 10–13). These results suggest that the RBPs that recognize the IL-3 ARE-rich region are specific and different from the RNA binding activities recognizing the hIL-3 3'-UTR Up and Down flanking regions.

Next, EMSA analyses were performed to assess whether the hIL-3 ARE-rich sequence is recognized by specific RBP complexes in Jurkat cells. As shown in **Fig. 2C** (lane 7), the hIL-3 ARE-rich region is also recognized by RBPs in human T-cells. In contrast to HeLa cells, no specific shift was noticeable for RNAs corresponding to the hIL-3 Up and Down flanking regions (**Fig. 2C**, lanes 6 and 8). Once again, no RNA binding activities were detected with the control-RNA in Jurkat cells (**Fig. 2C**, lane 5). The addition of proteinase K to the binding reactions abolished the shifts indicating that the complexes are dependent on RNA-protein interactions (**Fig. 2D**).

**Figure 3:**
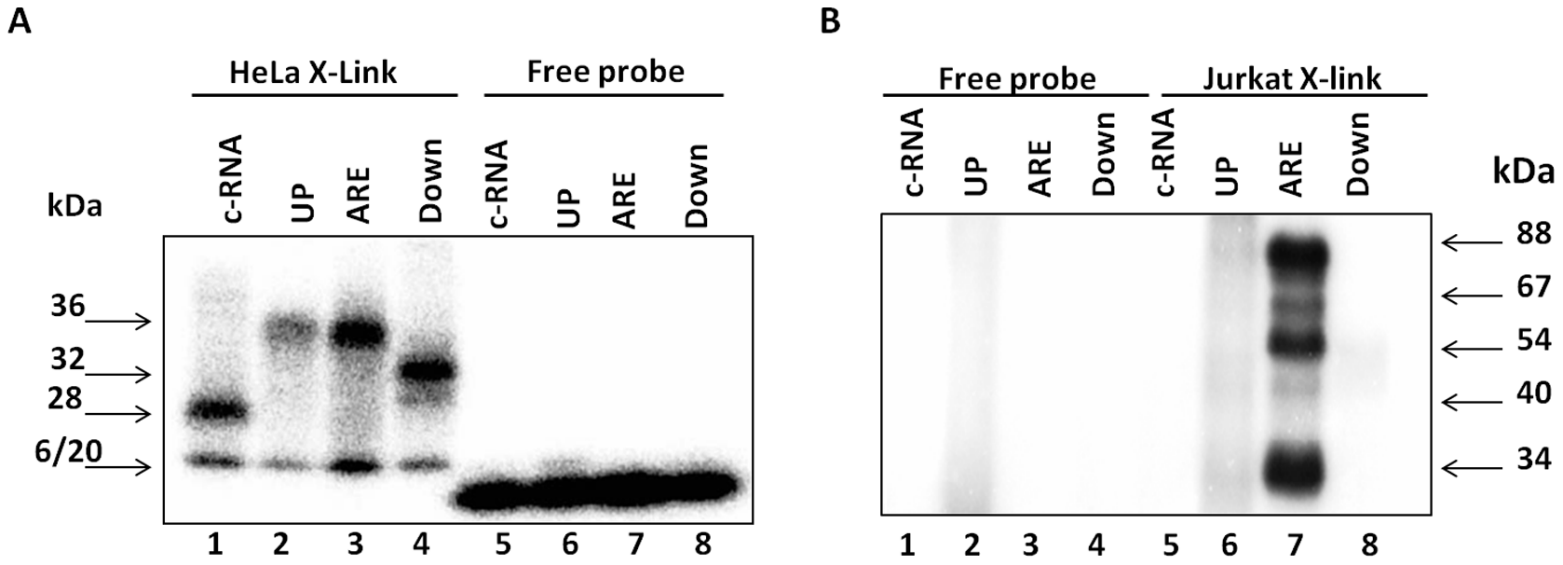
RNA-binding proteins ranging from 34-88 kDa recognize the hIL-3 ARE-rich region. (A) IL-3 Up, ARE and Down radiolabeled RNAs were incubated with HeLa cytoplasmic protein extracts (lanes 2–4) and the RNA-protein interactions were UV cross-linked. Samples were subjected to RNase digestion and to SDS-10% polyacrylamide gel electrophoresis. (B) UV-cross linking assays were also performed with Jurkat cytoplasmic cell extracts. IL-3 Up, ARE and Down radiolabeled RNAs were incubated with Jurkat cytoplasmic protein extracts (lanes 6-8).

To estimate the molecular masses of the RBPs that recognize the hIL-3 ARE-rich region, UV-cross linking assays were performed with both HeLa and Jurkat cytoplasmic protein extracts. In HeLa cells, the hIL-3 ARE-rich and Up regions are recognized by a 36 kDa RNA binding protein (**Fig. 3A**, lanes 2–3). The interaction of this RBP with the ARE-rich region appears to be stronger compared to its interaction with the Up region. In contrast, the Down region associates with a 32 kDa protein (**Fig. 3A**, lane 4) and the c-RNA probe is recognized by a 28 kDa protein that does not interact with the hIL-3 3'-UTR (**Fig. 3A**, lane 1). Furthermore, UV cross-linking assays with cytoplasmic extracts from Jurkat cells revealed five RBPs that recognized the hIL3-ARE-rich region with approximate molecular masses of 88, 67, 54, 40 and 34 kDa (**Fig. 3B**, lane 7). Interactions between RBPs and the Up or Down flanking regions were not detected in T cells. Collectively, these data suggest that the ARE-rich region present within the hIL-3 3'-UTR is recognized by specific RBPs in both HeLa and Jurkat T-cells.

### HuR and TIA-1 are components of the RBP complexes that recognize the hIL-3 ARE-rich region

**Figure 4:**
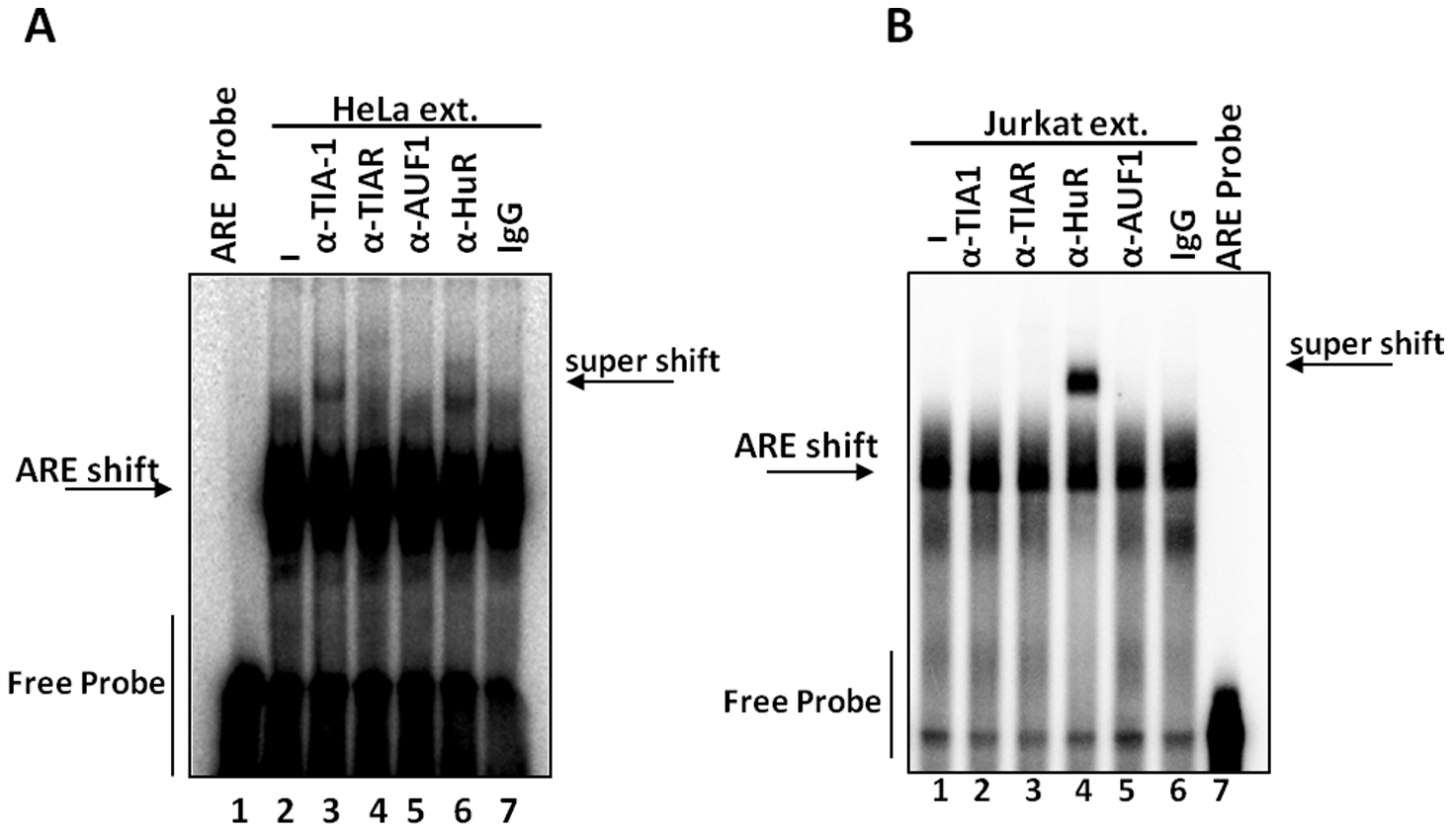
The hIL-3 ARE-rich region is recognized by HuR and TIA-1 ARE-BPs. (A) The ^32^P-labeled IL-3 ARE-rich sequence was incubated with HeLa cytoplasmic protein extracts and TIA-1, TIAR, AUF-1 and HuR antibodies. Non-immune goat serum (IgG) antibody was used as a negative control (lane 7). IL-3 ARE incubated with the HeLa cytoplasmic extract without antibody addition was used as an additional control in the analysis (lane 1). (B) Jurkat cytoplasmic protein extracts were also used in the EMSA super shift assays.

Previous studies have demonstrated that the ubiquitously expressed ARE-BP HuR recognizes the murine IL-3 ARE and regulates its expression at the post-transcriptional level [25], [37]. To determine whether HuR binds to the hIL-3 ARE-rich region, EMSA super-shift assays were performed with cytoplasmic extracts obtained from HeLa and Jurkat cells. As shown in **Figs. 4A and B**, HuR is a component of the hIL-3 ARE-rich RNA sequence/protein complex both in HeLa and T-cells. Based on the results described in **Fig. 3**, antibodies against other previously identified ARE-BPs whose molecular masses ranged between 34-88 kDa (e.g.TIA-1, TIAR and AUF1) were also included in this supershift analysis. Interestingly, the translational repressor TIA-1 is capable of recognizing the hIL-3 ARE-rich region only in HeLa cells. These results indicate that HuR and TIA-1 (at least in HeLa cells) are components of the RBP complexes that recognize the hIL-3 ARE-rich region.

### T-cell activation influences the association of specific RBP complexes that recognize the hIL-3 ARE-rich region

**Figure 5:**
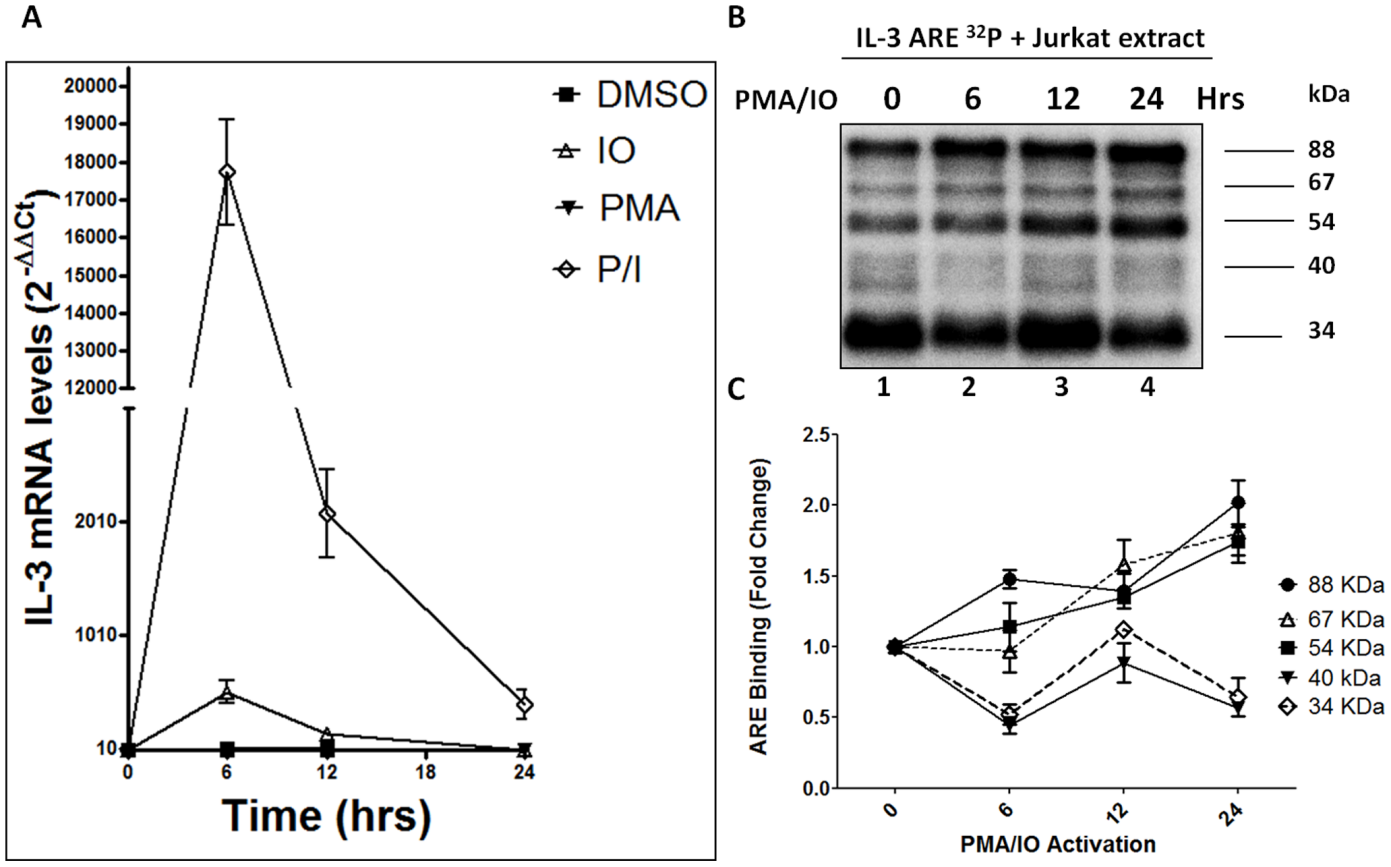
T cell activation regulates the ARE-BPs that recognize the hIL-3 ARE-rich region. (A) IL-3 endogenous mRNA levels were measured after T cell activation with Ionomycin (IO) and/or PMA at 0, 6, 12 and 24 hrs. Activation with DMSO was used as a negative control. (B) UV-cross linking assays were performed using the ^32^P labeled IL-3 ARE-rich RNA sequence incubated with 10 μg of activated Jurkat cytoplasmic protein extracts. (C) Graphic representation for ARE-binding quantification of the results obtained in UV-cross linking assays with activated Jurkat cytoplasmic extracts. Values represent mean ± standard error of the mean (SEM) from two experiments. Fold changes were normalized to 0 hours of T cell-activation with PMA/Ionomycin.

IL-3 is produced by T-cells *via* the TCR/CD3 signal transduction pathway [14]. T-cell activation via the TCR/CD3 pathway can be mimicked with PMA and Ionomycin, which activate protein kinase C and increase calcium influx [14]. Since T-cell activation is essential for hIL-3 expression [13], [14], we evaluated whether the hIL-3 ARE-BP complexes are modulated during Jurkat cells activation with PMA/IO. To validate the Jurkat cell activation protocol, we measured hIL-3 mRNA levels after 0, 6, 12 and 24 hours of incubation in the presence of vehicle (DMSO), IO, PMA or PMA/IO. As shown in **Fig. 5A**, T-cell activation with PMA and vehicle did not increase hIL-3 mRNA levels. Addition of IO demonstrated a slight increase of IL-3 mRNA levels at 6 hours. In contrast, activation with PMA/IO showed that hIL-3 mRNA levels reached maximum expression (35-fold accumulation) at 6 hrs followed by a gradual decline. These results confirm previous studies establishing the requirement of both phorbol esters and a calcium ionophore for optimal IL-3 expression in human T-cells [13], [14].

To determine whether T-cell activation modulates the interaction between hIL-3 ARE-rich region and the RBP complexes, UV cross-linking assays were carried out using labeled hIL-3 ARE RNA incubated with Jurkat cytoplasmic extracts activated with PMA/IO during 0, 6, 12 and 24 hours. **Figs. 5B and C** show a gradual increase in the binding of the previously identified 88, 67 and 54 kDa proteins to the hIL-3 ARE-rich region during T-cell activation. In contrast, the 34 and 40 kDa RBPs showed a fluctuation in their binding patterns towards the hIL-3 ARE. These results suggest that the RBP complexes associated with the hIL-3 ARE-rich region display a dynamic temporal association during T-cell activation.

### HuR interaction with the hIL-3 ARE-rich region is modulated during T-cell activation

**Figure 6:**
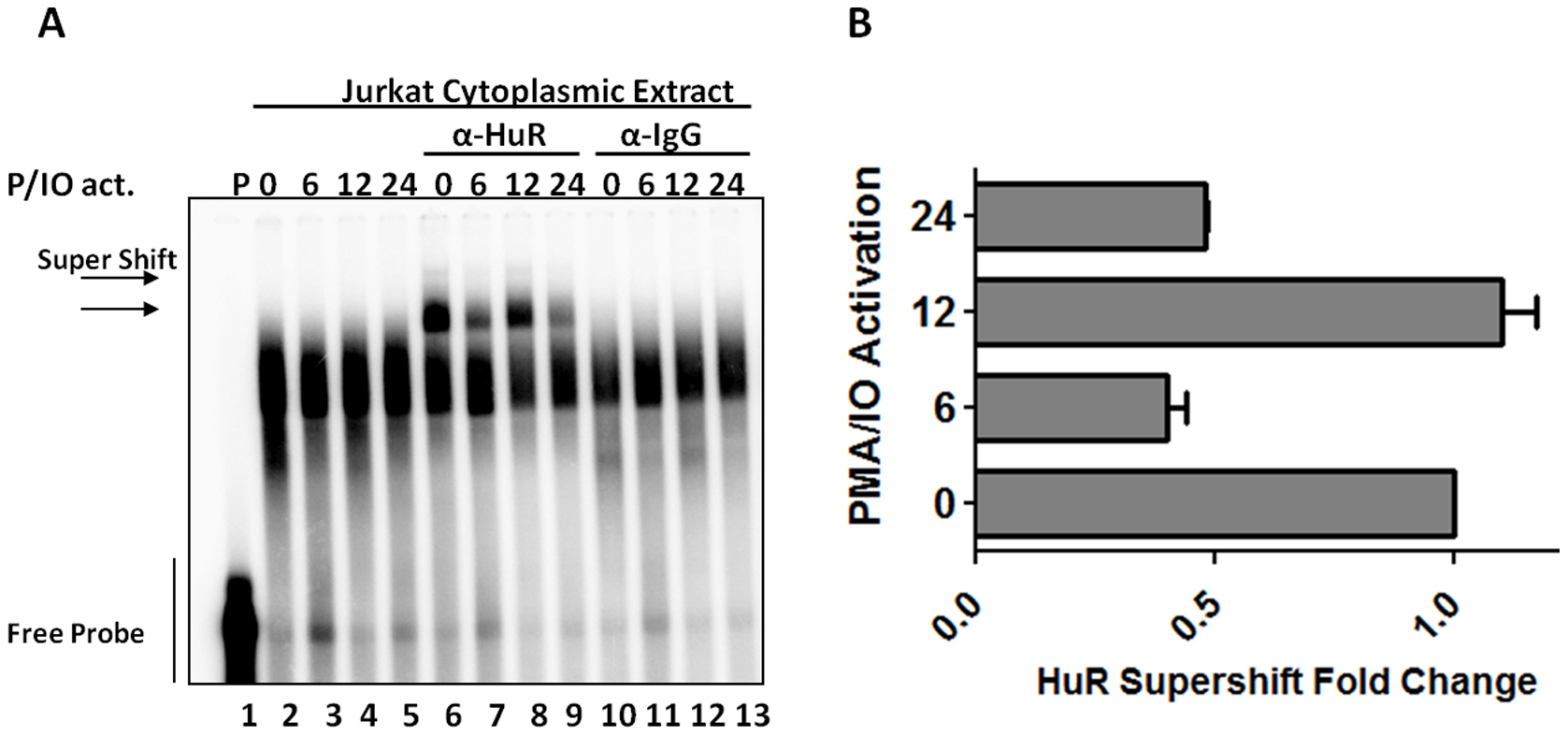
T cell activation modulates HuR binding towards the hIL-3 ARE-rich region. (A) HuR EMSA supershift analysis was carried out with Jurkat cytoplasmic extracts activated at 0, 6, 12 and 24 hours. (B) Graphic representation of HuR supershift quantification during T cell activation. Values represent mean ± standard error of the mean (SEM) from two experiments. Fold changes were normalized to 0 hours of T cell-activation with PMA/Ionomycin.

The observation that association of the RBP complexes with the hIL-3 ARE is dynamic during T-cell activation (**Figs. 5B and C**) suggested the possibility that the interaction between HuR and the hIL-3 ARE is altered during stimulation. Also, previous studies have shown that HuR is predominantly a nuclear protein that can be redistributed to the cytoplasm during T-cell activation, viral infection or in response to cellular stress [38]–[40]. To further assess the interaction between HuR and the hIL-3 ARE-rich region during T-cell activation, HuR supershift assays were performed with Jurkat cytoplasmic protein extracts activated with PMA/IO. As shown in **Figs. 6A and B**, HuR exhibited an association pattern consistent with the 34 kDa protein previously characterized in **Figs. 5B and C**, implying a dynamic association of HuR with the hIL-3 ARE-rich region during T-cell activation.

**Figure 7:**
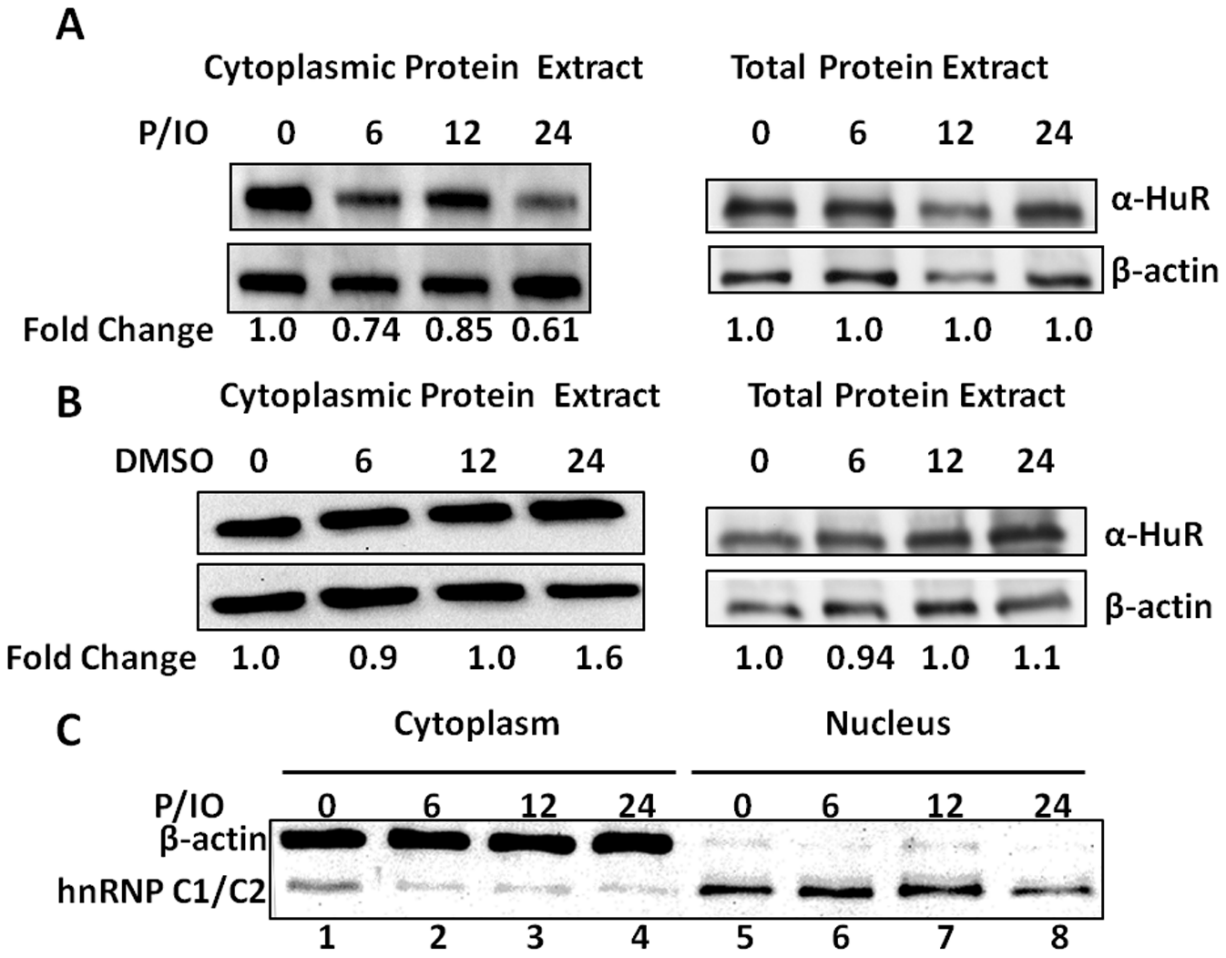
T cell activation modulates HuR spatial protein concentration. (A-B) Cytoplasmic and total protein extraction of Jurkat cells activated with DMSO and PMA/Ionomycin (P/IO) during 0, 6, 12 and 24 hours were used to perform a HuR immunoblotting. (C) β-actin and hnRNP C1/C2 protein distribution was determined in Jurkat cytoplasmic and nuclear protein extracts. Fold changes were normalized to 0 hours of T cell-activation.

To determine whether variations in HuR protein levels are responsible for changes in the interaction between HuR and the hIL-3 ARE-rich region during T-cell activation, HuR cytoplasmic levels were measured and compared to total HuR protein levels. As revealed by Western blot analysis, HuR cytoplasmic levels correlated with its dynamic association to the hIL-3 ARE-rich region (**Fig. 7A**). Vehicle (DMSO) addition at various time points showed a different HuR cytoplasmic accumulation pattern (**Fig. 7B**). Notably, HuR total protein levels remained constant after T-cell activation and vehicle addition (**Figs. 7A and B**). As fractionation controls, immunoblotting of β-actin and hnRNP C1/C2 demonstrated an adequate nuclear/cytoplasmic fractioning of the extracts (**Fig. 7C**). Collectively, the data suggest that changes observed in the interaction between HuR and the hIL-3 ARE-rich region during T-cell activation are exerted by alteration of HuR cytoplasmic levels that correlate with its redistribution from the nucleus to the cytoplasm upon stimulation.

### HuR plays a role in the hIL-3 3'-UTR-mediated regulation in human T-cells

**Figure 8:**
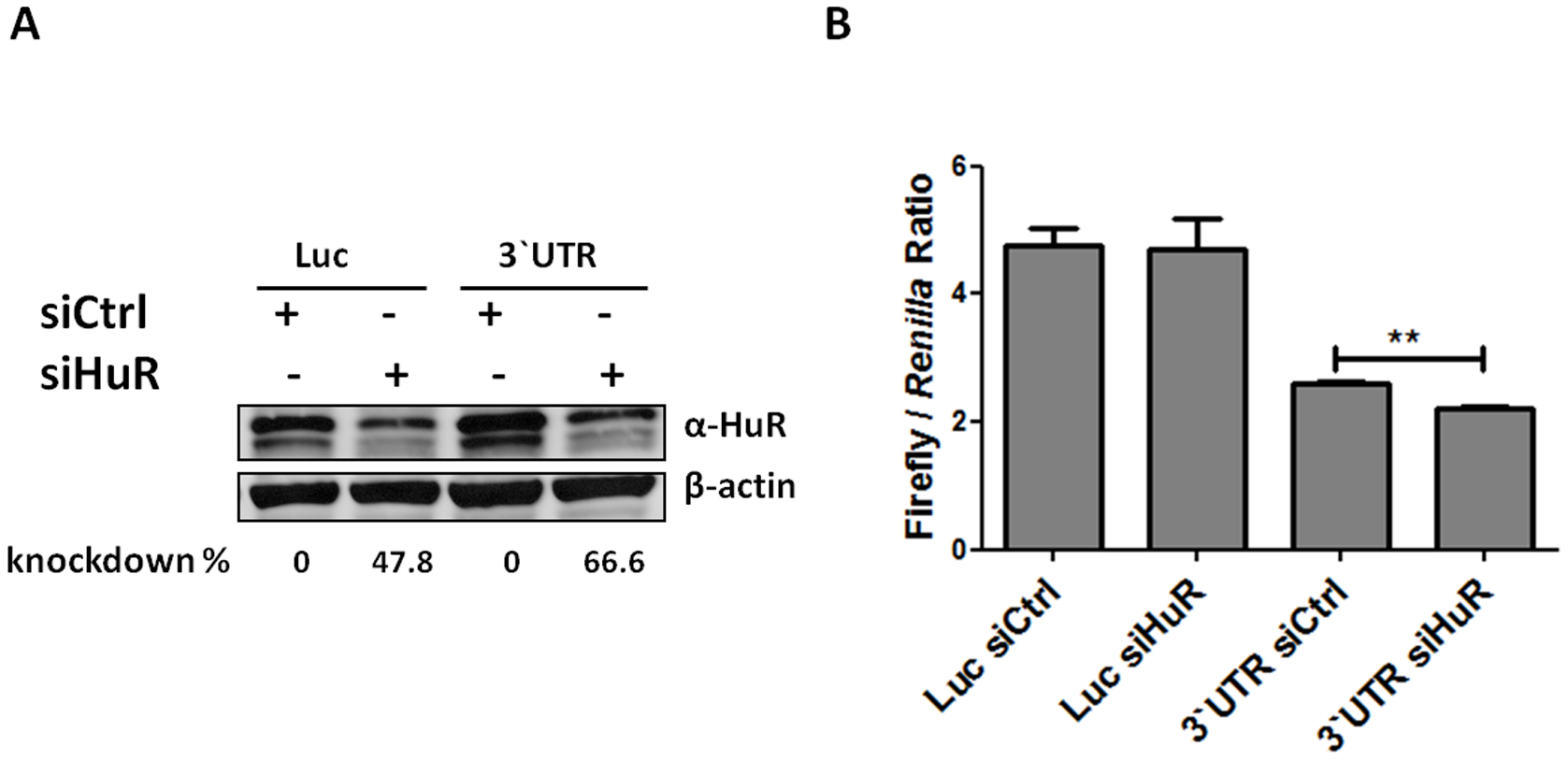
Knockdown of HuR increases the repression exerted by the IL-3 3'-UTR. Jurkat cells were co-transfected with the firefly reporter constructs (Luc and 3’UTR) and siRNA against HuR (siHuR) or control non-targeting siRNA (siCtrl). (A) Total protein extracts were used in Western blot analysis for HuR. β-actin was used as a loading control. (B) 48 hrs post-transfection, cells were harvested and luciferase activities were measured. Two-tailed *t*-tests were used for statistical analysis. The asterisk indicates a statistical significant (*P*<0.05) result when compared to the siCtrl.

Our previous results demonstrated an interaction between HuR and the hIL-3 ARE-rich region (**Fig. 4** and **6**). To test whether knockdown of HuR influences the expression of heterologous luciferase reporters, Jurkat cells were co-transfected with a siRNA directed against HuR and either with a luciferase construct harboring the entire hIL-3 3'-UTR or a control luciferase vector lacking the hIL-3 3'-UTR. After forty-eight hours, Western blot analysis revealed that transient transfection of HuR siRNA reduced HuR abundance by approximately 60%, compared with control siRNA-transfected cells (**Fig. 8A**). Also, the activity of the reporter chimeras was analyzed by dual luciferase assays. As shown in **Fig. 8B**, knockdown of HuR decreased the luciferase activity (1.2-fold) of the heterologous reporter harboring the hIL-3 3'-UTR whereas the activity of the luciferase chimera lacking the hIL-3 3'-UTR was unaltered by HuR silencing. These results further suggest a role for HuR in the 3'-UTR-mediated post-transcriptional regulation of IL-3 in human T-cells.

## Discussion

In this study, we demonstrated that the human IL-3 3'-UTR is involved in translational repression in both HeLa and Jurkat T-cells (**Fig. 1**). We went on to show that in Jurkat T-cells the 3'-UTR-mediated translational repression of hIL-3 is abolished by deletion of the region. In contrast, the hIL-3 ARE-rich region deletion only showed a partial regulatory effect in HeLa cells. We also identified specific RBP complexes that recognize the ARE-rich region within the IL-3 3'-UTR in both transformed human cell lines (**Figs. 2** and **3**). An EMSA supershift analysis (**Fig. 4**) identified TIA-1 (only in HeLa cells) and HuR (both in HeLa and Jurkat T-cells) as components of the RBP complexes that interact with the hIL-3 ARE-rich region. Given that IL-3 is endogenously expressed in T-cells, the differences observed in translational repression and the composition of the RBPs that recognize the IL-3 ARE-rich region might be associated with cell type specificity. Indeed, previous studies have demonstrated that the ARE-BP-mediated post-transcriptional regulatory pathway can vary among different cell lines [16]. For example, AUF-1 promotes mRNA decay of a β-globin reporter harboring the *c-fos* ARE in K562 pro-erythroblast cells while it stabilizes a reporter transcript in NIH-3T3 fibroblast cells [16], [41], [42]. In addition, TIA-1 represses TNF-α expression in macrophages but not in T-cells [17]. According to the current model, the activity of ARE-BPs and their relative expression levels in a particular cell can influence mRNA stability and/or translation of specific ARE-containing transcripts.

Previous studies have demonstrated that IL-3 expression is regulated upon T-cell activation with antigens, mitogens and phorbol esters [13], [14]. Moreover, Moroni and colleagues have shown that the ARE regulates mRNA stability of the murine IL-3 transcript during T-cell activation [15], [43]. In contrast, our studies with human T cells at resting state suggest a translational repression role for the hIL-3 ARE-rich region (**Fig. 1**). Perhaps, the hIL-3 ARE-rich region exerts a dual regulatory role in mRNA stability and/or translation upon T-cell activation. Although AREs from human and murine IL-3 are approximately 85% identical, their 3'-UTRs display only 51% identity (data not shown). Therefore, the particular arrangement of ARE motifs within the different 3'-UTRs might influence the spectrum of RBP complexes (translational regulators versus mRNA stability factors) that recognize these *cis*-acting elements. Further investigation of these apparent discrepancies will be required to understand the precise functional role of AREs in the post-transcriptional control of the IL-3 transcript.

HuR has been previously characterized as an ubiquitously expressed ARE-BP with dual function in the regulation of mRNA stability and translational control of various ARE-containing transcripts [21], [44]–[47]. Raineri *et al*. demonstrated that HuR mediates the post-transcriptional control exerted by the murine IL-3 ARE [37]. This analysis showed that HuR siRNA knockdown in fibrosarcoma cells (HT1080) decreases protein expression of a heterologous reporter harboring the mIL-3 ARE. Using an *in-vitro* RNA-protein binding assay, Furneaux and colleagues further demonstrated that HuR interacts with the murine IL-3 ARE [48]. However, no evidence for a physical interaction between HuR and the human IL-3 3'-UTR has been reported. In this study, we demonstrated that HuR is a component of the RBP complexes that recognize the ARE-rich region within the hIL-3 3'-UTR both in HeLa and Jurkat cells (**Figure 4**). Consistent with previous studies obtained with murine IL-3 [37], we showed that HuR knockdown in human T-cells decreases the luciferase activity of a chimeric reporter harboring the human IL-3 3'-UTR (**Fig. 8**). Together, these results suggest that HuR, at least in Jurkat cells at resting state, promotes protein expression by interacting with *cis*-acting elements (e.g., AREs) present within the human IL-3 3'-UTR. Previous studies have demonstrated that HuR-mediated post-transcriptional control is regulated by post-translational modifications (*e.g.* phosphorylation) and it can act as a positive and/or negative regulator of gene expression [49]-[53]. At present, the mechanistic details of this HuR-mediated post-transcriptional regulation of hIL-3 are still missing. Additional studies are required to further understand whether HuR influences IL-3 protein levels by altering mRNA stability and/or translation in human T-cells upon T cell activation.

Previous studies have demonstrated that HuR over-expression in macrophages activated with lipopolysaccharides (LPS) reduces protein levels of proinflammatory factors such as TNF-α and COX-2 [51]. Since IL-3 expression is coordinately regulated in an activation-dependent manner, we also analyzed the interaction between HuR and the hIL-3 ARE-rich region upon T-cell activation using PMA and Ionomycin. As demonstrated in the EMSA supershift analysis, HuR shows a fluctuation in its binding pattern towards the hIL-3 ARE-rich region upon T-cell activation (**Fig. 6**). Interestingly, the variations observed in HuR protein cytoplasmic levels correlated with HuR binding to the hIL-3 ARE-rich sequence (**Fig. 7**). Analysis of total cell extracts upon T-cell activation showed that HuR protein levels were constant throughout the course of the experiment (**Figs. 7A & 7B**). These results suggest that variations in the interaction of HuR and the hIL-3 ARE-rich region upon T-cell activation can be modulated by changes in HuR spatial localization. Previous studies have demonstrated that HuR shuttles from the nucleus to the cytoplasm after infection of the Sindbis virus (SinV) [40]. In addition, HuR is rapidly translocated to the cytoplasm in response to oxidative stress and at early stages of T-cell activation [54]–[57]. Importantly, the increase of HuR protein in the cytoplasm correlates with a transient expression of target mRNAs encoding early-response transcription factors and cell cycle modulators including: c-Fos, Egr-1, cyclin A and p21 [57], [58]. Our results are consistent with previous reports suggesting that the rapid translocation of HuR messenger ribonucleoprotein complexes (mRNPs) plays a role in the post-transcriptional control of early response factors during early stages of T-cell activation.

In summary, we have presented evidence for a translational repression role for the human IL-3 3'-UTR in transformed cell lines (HeLa and Jurkat cells). The ARE-BP HuR recognizes the hIL-3 ARE-rich region in T cells and plays a role in the post-transcriptional control of a heterologous reporter harboring the IL-3 3'-UTR. Upon T cell activation, we observed changes in both HuR binding to the hIL-3 ARE-rich region and HuR cytoplasmic levels. Since IL-3 is an early response cytokine transiently expressed at the early stage of T-cell activation and its aberrant expression has been linked to different leukemias [59], understanding how HuR protein regulates IL-3 mRNA expression at the post-transcriptional/translational level in T lymphocytes will be critical for developing effective therapies against cancer and other human blood disorders.
