# Correction: The Role of HuR in the Post-Transcriptional Regulation of Interleukin-3 in T Cells

**DOI:** 10.1371/journal.pone.0106296

**Published:** 2014-08-15

**Authors:** The PLOS ONE Staff

Information is missing from the Funding section. The correct funding information is as follows: This work was supported by grants from the National Institutes of Health to CIG (GM008102-3052, KO1 HL-04355-05, U54 CA96297, FIPI, PES-UPR), JGF (PR-LSAMP and RISE Award 2R25GM61151), MHP and MM (R25GM061838). This publication was made possible by Grant P20 RR 016174 from the National Center for Research Resources, a component of the National Institutes of Health. The funders had no role in study design, data collection and analysis, decision to publish, or preparation of the manuscript.
